# Patient-Controlled Analgesia and Peripheral Nerve Block Increase the Risk of Post-operative Urinary Retention after Total Knee Arthroplasty in Asian Patients

**DOI:** 10.5704/MOJ.2407.006

**Published:** 2024-07

**Authors:** YH Kwan, KG Tan

**Affiliations:** Department of Orthopaedic Surgery, Tan Tock Seng Hospital, Singapore

**Keywords:** urinary, retention, knee, arthroplasty, analgesia

## Abstract

**Introduction:**

Post-operative urinary retention (POUR) is a common complication after total knee arthroplasty (TKA) and may result in severe complications such as urinary tract infection and deep joint sepsis, leading to prolonged hospital stay and increased medical costs. Hence a retrospective study was performed to identify the incidence and modifiable factors associated with POUR after elective TKA in Asian patients with the aim to prevent POUR and its undesirable consequences.

**Materials and Methods:**

The medical records of 496 consecutive patients who underwent elective TKA between 1 August 2017 and 30 July 2018 were reviewed. There were 154 male (31.0%) and 342 female (69.0%) patients with an average age of 68 years old. The incidence of POUR was analysed with respect to various modifiable and non-modifiable risk factors, including patient demographics, medical comorbidities, duration of surgery, type of intra-operative anaesthesia and post-operative analgesia and early initiation of physiotherapy using univariate and multivariate analyses.

**Results:**

A total of 120 (24.2%) of the 496 patients who underwent elective TKA developed POUR. The odds of a patient with patient-controlled analgesia (PCA) and peripheral nerve block (PNB) developing POUR were 4.2 times and 4.7 times that of a patient without PCA and PNB, respectively. Age, male gender and type of anaesthesia were not found to be significant.

**Conclusion:**

In our study population, the incidence of POUR after elective TKA was 24% with major modifiable risk factors being associated with the use of PCA and PNB as post-operative anaesthesia. POUR can have deleterious effects thus alternative post-operative analgesia should be considered.

## Introduction

Post-operative urinary retention (POUR), defined as the inability to void after surgery^1^, is a common surgical complication with an incidence of 10.7-77.8% after total joint arthroplasty^2^. Apart from causing patient discomfort and anxiety on top of post-operative pain^2^, POUR has potentially severe consequences namely bladder dysfunction and urinary tract infection (UTI) which can in turn lead to a longer hospital stay as well as prosthetic joint infection (PJI) through haematogenous seeding^3^.

The literature has described a number of potential risk factors for POUR following lower limb arthroplasty in general, in other words both TKA and total hip arthroplasty (THA), such as older age, previous urinary retention, hypertension, diabetes, benign prostatic hypertrophy, excessive intra-operative fluids, and epidural anaesthesia^1-2,4^. As the prevalence of knee osteoarthritis in Singapore is disproportionately higher than that of hip osteoarthritis in view of the majority Asian population^5^, and the demographics of patients undergoing total knee and total hip arthroplasties differ^6^, we believe it would be beneficial to identify and address risk factors for POUR specifically after TKA.

Hence the aim of this study was to determine the incidence of POUR, its impact on outcomes of TKA, and analyse and modify any modifiable risk factors associated with POUR.

## Materials and Methods

**Fig. 1:**
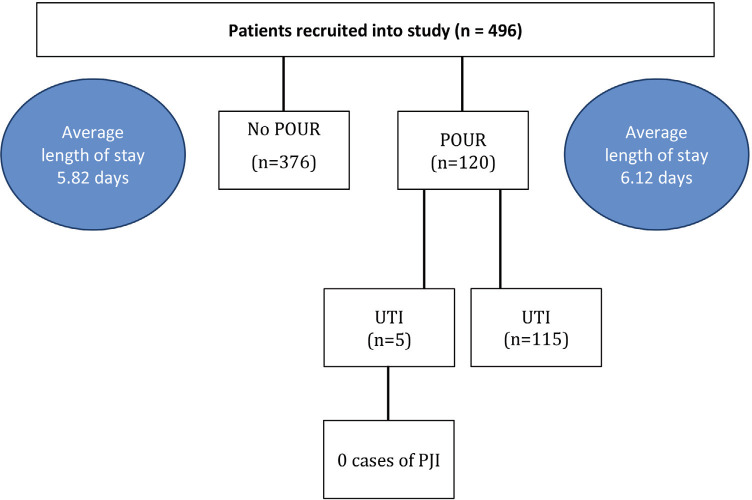
The flowchart shows the outcomes of the study. (POUR: post-operative urinary retention, UTI: urinary tract infection, PJI: prosthetic joint infection).

Four-hundred and ninety-six consecutives primary TKA patients admitted to a dedicated joint replacement ward in a tertiary hospital over the period of 1st August 2017 to 30th July 2018 were included. Patients who had IDC inserted pre-operatively or intra-operatively for any reason, for example, chronic neurogenic bladder, were excluded from the study. All data including the primary outcome of POUR as well as secondary outcomes such as the length of hospital stay and the development of UTI and PJI were prospectively collected as part of the institution’s registry database. Retrospective analysis of relevant modifiable and non-modifiable risk factors were performed and shown in Table I and Fig. 1.

**Table I:** Patient demographics and medical comorbidities.

Demographic/medical comorbidity	Number of patients
Sex (male / female)	154 (31%) / 342 (69%)
Average age (years)	68.46
Diabetes mellitus	114 (23%)
Hypertension	342 (69%)
Hyperlipidaemia	289 (58%)
Ischemic heart disease or congestive cardiac failure	26 (5%)
Inflammatory arthritis	10 (2%)
Benign prosthetic hypertrophy	17 (3%)
Depression	6 (1%)

The modifiable risk factors were selected based on prevailing understanding that they may influence POUR. The duration of surgery and post-operative analgesia have been described to influence the occurrence of POUR in previous studies^7^. Early mobilisation may be beneficial in restoring physiological functions of the body post-operatively including micturition. Smoking has been reported to potentially accelerate atherosclerosis and in turn decrease bladder perfusion by pelvic blood vessels and hence increase the risk of POUR^8^.

As there is no current standardised definition for POUR, we defined POUR either clinically as the inability to voluntarily void within 6 hours post-operatively and thus requiring indwelling catheter (IDC) insertion, or quantitatively as having a post void residual volume (PVRU) of more than 300mL and hence requiring IDC insertion. According to physiological studies of the bladder, a sensation of fullness should be felt at a bladder volume of 300mL^7^. As part of a standardised post-operative nursing protocol for patients post-TKA house in the same arthroplasty ward, a bladder scan was performed on all patients who could not void within 6 hours post-operatively, as well as those who could void but complained of lower abdominal discomfort with or without bladder distension on palpation. The clinical findings and PVRU were documented in the patients’ electronic case notes by the attending staff nurse or doctor who examined the patient. Urine culture was performed for all patients who required IDC insertion for POUR upon the insertion of the IDC and UTI was defined as a urine culture positive for bacterial growth. PJI would have been diagnosed only if the patients had developed clinical signs and symptoms of joint infection and fulfilled the Musculoskeletal Infection Society’s (MSIS) definition of PJI.

Univariate and multivariable logistic regression analysis were performed, using [IBM SPSS Statistics for Windows, Version 23.0]. Statistical significance was determined using t-test and non-parametric tests. A P-value of less than 0.05 was considered statistically significant.

## Results

There were 154 male (31.0%) and 342 female (69.0%) TKA patients with a mean age of 68 years old (range, 51-89 years) and a wide range of co-morbidities. Hypertension was the most common comorbidity present, followed by hyperlipidaemia and diabetes. Around 10% of the male patients had a pre-existing diagnosis of benign prostatic hypertrophy (BPH).

Out of 496 patients, 124 patients underwent general anaesthesia (GA)), and 372 underwent spinal anaesthesia (SA). All General anaesthesia (GA) involved the use of opioids such as morphine and all spinal anaesthesia (SA) involved the use of bupivacaine. A total of 143 of these patients received Patient controlled analgesia (PCA), consisting of either morphine or fentanyl, while the remaining 353 patients received a peripheral nerve blockade as post-operative analgesia.

A total of 120 patients developed POUR, giving an incidence of 24.2%. All patients who fulfilled the criteria for POUR received indwelling catheters. After applying univariate and multivariable analyses (Table II and Table III), PCA (P-value = 0.025, Odds-ratio [OR] = 4.23; (1.31 – 16.89)) and PNB (p = 0.016, Odds-ratio [OR] = 4.70; (1.47 – 19.0)) were found to be the only significant modifiable risk factors for POUR. A patient with PCA were 4.2 times (95% Confidence Interval: 1.31 – 16.89) more likely to develop POUR than a patient without PCA (p = 0.025), while a patient with PNB were 4.7 times more likely to develop POUR than a patient without PNB (95% Confidence Interval = 1.47 – 19.0, p = 0.016). All other risk factors including male gender, age, history of Benign Prostate Hyperplasia (BPH), and type of anaesthesia were not significantly associated with POUR.

**Table II:** Univariate analysis of modifiable and non-modifiable factors for POUR.

Variable	Odds ratio (95% C.I.)	P value
Sex		
Male	1.0	-
Female	0.788 (0.56 – 1.11)	0.168
Age	1.02 (0.99 – 1.05)	0.199
Comorbidities		
Diabetes mellitus	1.10 (0.70 – 1.73)	0.694
Hypertension	1.25 (0.80 – 1.96)	0.335
Hyperlipidaemia	0.917 (0.62 – 1.37)	0.683
Ischemic heart disease or congestive cardiac failure	0.583 (0.20 – 1.71)	0.332
Inflammatory arthritis	0.780 (0.18 – 3.43)	0.755
Benign prosthetic hypertrophy	1.75 (0.74 – 4.16)	0.207
Depression	1.58 (0.50 – 5.03)	0.447
Comorbidity		
Smoker	0.894 (0.58 – 1.38)	0.623
Type of intra-op anaesthesia		
Spinal	1.0	-
GA	1.5 (0.98 – 2.30)	0.0621
Surgery duration (minutes)	1.01 (1.00 – 1.02)	0.0990
Post-operative PCA	0.964 (0.63 – 0.68)	0.876
Post-operative PNB	1.35 (0.85 – 2.15)	0.208
Physiotherapy at POD 0	1.63 (0.90 – 2.97)	0.110
Haemoglobin drop (g/dL)	1.10 (0.89 – 1.36)	0.383
Length of stay (days)	1.04 (0.97 – 1.12)	0.291

*95% C.I.: 95% confidence interval, GA: general anaesthesia, PCA: patient-controlled analgesia, PNB: peripheral nerve block, POD: post-operative day

**Table III:** Multivariable analysis of modifiable and non-modifiable factors for POUR.

Variable	Odds ratio (95% C.I.)	P value
Sex		
Male	1.0	-
Female	0.75 (0.46 – 1.22)	0.238
Age	1.02 (0.99 – 1.06)	0.144
Comorbidities		
Diabetes mellitus	0.86 (0.50 – 1.47)	0.594
Hypertension	1.39 (0.83 – 2.38)	0.216
Hyperlipidaemia	0.85 (0.52 – 1.40)	0.533
Ischemic heart disease or congestive cardiac failure	0.515 (0.14 – 1.46)	0.252
Inflammatory arthritis	0.751 (0.10 – 3.66)	0.743
Benign prosthetic hypertrophy	1.23 (0.39 – 3.61)	0.706
Depression	1.38 (0.18 – 7.44)	0.718
Comorbidity		
Smoker	0.61 (0.08 – 2.76)	0.553
Type of intra-op anaesthesia		
Spinal	1.0	-
GA	1.56 (0.96- 2.53)	0.072
Surgery duration (minutes)	1.00 (1.00 – 1.01)	0.147
Post-operative PCA	4.23 (1.31 – 16.89)	0.025
Post-operative PNB	4.70 (1.47 – 19.0)	0.016
Physiotherapy at POD 0	1.48 (0.80 – 2.87)	0.229
Haemoglobin drop (g/dL)	1.07 (0.85 – 1.35)	0.555
Length of stay (days)	1.01 (0.93 – 1.09)	0.785

*95% C.I.: 95% confidence interval, GA: general anaesthesia, PCA: patient-controlled analgesia, PNB: peripheral nerve block, POD: post-operative day

Five patients (2 males and 3 females) developed UTI as evidenced by urine cultures positive for bacterial growth. All five patients completed a five-day course of oral antibiotics for treatment of UTI. There were no cases of superficial or deep wound sepsis, as well as PJI amongst the cohort of primary TKA patients. The mean length of stay of the entire group was 5.89 days with POUR patients staying an average of 0.4 days longer (p<0.05) (5.82 days compared to 6.12 days).

## Discussion

The incidence of POUR after TKA in this study is 24.2%. This study also demonstrates that the use of PCA or PNB as post-operative analgesia is significantly associated with an increased risk of POUR after TKA. Other non-modifiable and modifiable risk factors studied did not demonstrate a statistically significant association with POUR.

The incidence of POUR in this study of 24.2% is much higher than that of another local study done in 2007 by Lingaraj *et al* (8%)^9^, and a UK study done in 2008 by Kumar *et al* (19.7%)^10^, possibly due to our inclusion of patients who were unable to void spontaneously as well as those with post void residual volume of more than 300mL. The 2 studies were also comparatively smaller with fewer than 150 patients each. However, our incidence is comparable to the incidence of 22.9% reported in the study by Balderi *et al* when POUR is defined as the failure to void with a bladder volume of more than 500mL requiring catheterisation^11^. On the other hand, Bjerrergaard *et al* found that the incidence of POUR in a similar sample size of 474 fast-track TKA patients was 46.2^12^ but remarked that even within their study there was considerable variation in incidence amongst the four orthopaedic departments recruited, likely due to different thresholds for catheterisation. Balderi *et al* reported previously that the incidence of POUR in lower limb arthroplasty had a wide discrepancy amongst previous studies (between 0% and 75%) because of the large variation in the definition of POUR and criteria for catheterisation^13^. We believe that this is the main reason why our incidence of POUR is different from that of other studies as well. Bjerrergaard *et al* included a more subjective criterion for catheterisation whereby patients with symptomatic urinary retention was catheterised regardless of their bladder volume, which may have contributed to their higher incidence of POUR^12^.

This study showed that the use of PCA as post-operative analgesia increased the risk of POUR in post-operative TKA patients. The literature on the risk of developing POUR with PCA after joint replacement surgery is scarce. However, our findings concur with that of a few studies^14,15^. We postulate that this is likely due to the effects of opioids that are routinely employed in PCA in our institution, as systemic opioids work on spinal cord receptors that control the contractility of the detrusor muscle and it has been hypothesised that PCA produce a constant plasma opioid concentration with a prolonged effect on the bladder muscle^7^.

Interestingly, our study also found a significant risk of POUR associated with use of PNB although there should not be any direct pharmacological effect of the anaesthetic agent used in PNB on bladder function. Studies comparing PNB with other forms of post-operative analgesia such as epidural analgesia have also described the development of POUR after the use of PNB, albeit at a lower risk than epidural analgesia^16^. We postulate that the increased risk of POUR can be attributed to reduced mobility secondary to quadriceps paresis after a femoral nerve block, a consequence identified in Sharma *et al’s* study on the complications of femoral nerve block for TKA^17^. Alternatively, use of highly selective motor sparing PNB such as “hunter canal block” can be employed however this was not commonly practiced in our institution.

Our study found that the type of anaesthesia did not have a significant influence on POUR. This is despite the fact that GA and SA have been theorised to have known physiological effects on bladder tone and urethral sphincter control that can lead to urinary retention; GA by causing bladder atony by affecting the autonomic nervous system, and SA through blockade of afferent input from the bladder to the micturition centre^7^. Baldini *et al* reviewed a variety of anaesthetic factors associated with POUR after surgery (not limited to Orthopaedic surgeries)^7^, and found that long-acting spinal local anaesthetic was associated with the development of POUR in several studies, whereas GA resulted in a significantly lower incidence of POUR compared to spinal or epidural anaesthesia. Fernandez *et al* found that a significant number of male patients who underwent hip or knee arthroplasty developed POUR after receiving SA with intrathecal morphine compared to GA with femoral nerve block or SA with intrathecal fentanyl^18^. However, there was no significant difference amongst the different anaesthetic regimes in the female group. Griesdale *et al* also found that SA without morphine did not increase the risk of POUR in both males and females who underwent unilateral total hip or knee arthroplasty^19^, although males who received SA with morphine had higher odds of developing POUR compared to those who received GA. However, both studies did not account for prostate conditions in their male cohorts.

Interestingly, the theoretical risk factors for POUR, namely male gender and BPH, did not show a significant association in our study population after univariate and multivariable analysis. Recent large studies by Bjerregaard *et al* and Kort *et al* have also showed no gender significance^12,20^, which concurs with our finding that male gender is not significantly associated with POUR. Also, observational studies by Griesdale *et al* and Sung *et al* have identified that male patients in their studies may have had undiagnosed bladder outflow tract pathologies that predisposed them to POUR^19,21^. This did not appear to have been a confounder in our particular study. Our results showed that BPH did not significantly increase the risk of POUR. Most previous studies did not study BPH as an independent factor for POUR, rather, they had deduced that males were more likely to develop POUR because of bladder outlet obstruction pathologies like BPH9,19,21. Bjerregaard *et al* had also identified that the International Prostate Scoring System which measures the severity of BPH symptoms did not reveal any association between the severity of bladder outlet obstruction and the likelihood of requiring IDC insertion despite showing a significant association with POUR^12^. However, it is also possible that our patients with the diagnosis of BPH had been conservatively treated previously or were receiving ongoing treatment for BPH during our assessment.

A variety of methods to mitigate the risk of POUR after TKA have been suggested in the literature. Routine insertion of IDC for patients receiving PCA or PNB would be controversial. A meta-analysis in 2015 by Zhang *et al* that compared the rates of UTI and POUR after IDC or intermittent catheterisation after total joint arthroplasty showed that inserting an IDC for 24 to 48 hours post-operatively was superior to intermittent catheterisation in preventing POUR and did not increase the risk of UTI as well^22^. However, a more recent meta-analysis by Ma *et al* found that total hip and knee patients with IDC had a higher risk of UTI compared to those without, although the study was limited by the small number of randomised controlled trials and their differing criteria for POUR^23^. A systematic review by Jackson *et al* found that intra-operative epidural/pudendal nerve blockade, giving intramuscular drotaverine after spinal anaesthesia, early mobilisation and avoiding the use of morphine post-operatively were associated with a lower incidence of POUR across various types of surgeries^24^. However, the authors identified several limitations to the review including heterogenous study populations, anaesthetic and surgical techniques and criteria for POUR, and small number of studies that compared the same interventions. A literature review article in 2008 by Kotwal *et al* cited studies that suggest the use of pharmacological agents, namely Prazosin and Phenoxybenzamine, to relax the smooth muscles of the urethra and prostate and hence prevent POUR after total joint arthroscopy^2^. However, the authors acknowledged that the utility of those medications was limited by their potential side effects. Lastly, Enhanced Recovery After Surgery [ERAS®] protocols, which have been introduced in the management of post-TKA patients with the goals of minimising post-operative pain, encouraging early mobility and reducing length of stay and complication rates, have recommended the use of local infiltration analgesia (LIA) that is administered within the knee joint intra-operatively over PNB and PCA as it is able to provide equally efficacious pain relief in the absence of side effects that may result from PNB (motor blockade) or PCA (POUR, nausea, vomiting and sedation)^25^.

Our study concurs with avoiding the use of PCA and PNB in post-operative pain management in view that it increases the risk of POUR and hence disrupts recovery and increase length of hospital stay. Our institution has increased the utilisation of LIA together with multimodal oral analgesia as the mainstay of our post-operative pain management and significantly reduced the use of PCA and PNB in our TKA patients^26^. This may contribute to the reduction in the rate of POUR which can be explored in future studies.

One limitation of our current study lies in its retrospective and observational nature, bearing the bias of a retrospective study. The incidence of UTI may have been under-diagnosed due to the routine administration of perioperative antibiotic prophylaxis which is a broad-spectrum antibiotic such as cefazolin over a period of 24 hours. However, our study employed vigorous research methodology; having a large sample size obtained from a dedicated arthroplasty ward with standardised post-operative surgical and nursing protocols such that all patients with POUR are recorded accurately and managed in the same manner.

## Conclusion

The incidence of POUR after elective TKA in our local tertiary hospital’s joint replacement ward is 24.2%. The only significant modifiable risk factors identified with POUR were the use of PCA and PNB as post-operative analgesia. As POUR can prolong inpatient stay and potentially lead to serious consequences including UTI and even PJI, it would be prudent to avoid the use of PCA and PNB and instead consider alternative post-operative analgesia such as local infiltration analgesia that would avoid the need for invasive catheters and allow quicker mobilisation with the aim to reduce the incidence of POUR after TKA in Asian patients.
